# Sponge Gold

**Published:** 1853-10

**Authors:** 


*Sponge Gold.*—We have repeatedly called attention to this article, since the first sample of it was received. At the instance of the senior editor of this Journal, several experiments were instituted by the junior editor, to ascertain the method of making it.

It was found that a variety of plans would give a spongy, porous cake of gold which would readily weld. Indeed, gold in fine powder, as precipitated by the protosulphate of iron or oxalic acid, welds readily under pressure, a property which is also possessed by finely divided silver, though not in the same degree. The objection to employing it in this way, consists in its extreme division, the lightness of the powder, and the consequent great waste in the mouth.

A porous mass may be readily obtained by fusing together one part of gold and two or two-and-a-half of silver, running the alloy through a rolling mill, to reduce it to a thin ribbon, rolling this up into a spiral coil, and treating it with nitric acid; in short, going through all the manipulations of the well-known metallurgic operation of parting. Three doses of nitric acid are necessary, the first of a weak acid, the next of one of medium strength, and the third, of concentrated acid. This process, if properly conducted, dissolves out all the silver, with the exception of a very minute portion, and leaves the gold much purer and more adhesive than ordinary foil. The cornet, however, as taken out of the flask after the parting is completed, is very weak and frail, owing to the extreme porosity and great expansion of the gold. It cannot even be handled, it is so tender. It must, therefore, be carefully tilted into a crucible or some such vessel, and heated to orange redness in a muffle, taking care not to fuse it. When it is removed, it will be found to have contracted very considerably, to have become firmer, and to have assumed something of a golden hue, instead of the dull and almost coppery brown, it had before being heated. It is now no more than a roll of heavy foil, perforated in all directions with minute pores, occupying the place of the particles of silver which have been removed by the acid. This operation requires great care. An unskillful and inexperienced hand will leave so much silver in it, as seriously to interfere with its welding, and to give it a pale lemon yellow tint, or will destroy the cornet by reducing it to a mere powder. When well made, it should be a deep yellow; approaching a red, and its surface should have the appearance of deadened gold.

Another method in which gold can be brought to the condition of a porous cake, is simpler than the last. A solution of perfectly pure gold in aqua regia is evaporated to dryness, and then slowly heated to an orange red, at which it is to be kept a few minutes. There remains an irregularly porous cake, which welds with great facility.

In our last issue, we published the formula of the New York patentee, which gives a crystalline mass, or a lustreless sponge, according to the manner in which the operation is conducted. We refer our readers who desire to be informed on this subject, to our July number.

Some months since, the senior editor received a letter containing a specimen of sponge gold from Mr. Tomes, prepared by Mr. Barling, which was equal, if not superior, to any preparation of the kind he had used up to that time. He has recently received another letter from Mr. T., which he hastens to lay before the readers of the Journal. The preservation of the article in naptha, is an excellent idea. A perfectly pure surface is thus ensured, and it is not necessary, to inform our readers, how essential that is to the success of any attempts to weld gold.

We publish below that portion of Mr. Tomes' letter relating to this article.

37 Cavendish Square, London, *August 8th*, 1853.

*Dear Sir:*—Some months since I wrote to you, * * * and enclosed a specimen of the sponge gold prepared here. Since that time, the party who made the preparation has been actively engaged in its improvement. A few days since, he sent me a bottle of sponge gold far superior to any I had seen before. It is kept in naptha, and this is ignited and burnt off when you want to use gold. Treated in this manner, it is most extraordinarily adhesive, and readily works up into a solid plug; bit by bit is added, the fresh morsels readily uniting with that which has been compressed, it being necessary only to keep the surface dry and a little rough. I find it best to press the gold into the cavity with a tolerably large instrument, and then to use a pointed one, of course; burnishing in the progress of the filling is to be avoided. After completing the plugging and filing, the burnishing requires to be done with a light hand, otherwise, from the peculiar condition of the gold, the instrument becomes coated with the metal, and scratches, instead of polishing. I feel greatly interested in the ultimate results of the experiment; because, I think, the preparation intrinsically good, and because, I trust the maker may be remunerated for his labor and expenses. I give you his address. Mr. Barling, Jeweller, High Street, Maidstone, Kent, England.

Yours, faithfully, John Tomes.

